# Integrated Molecular and Functional Characterization of Cervical Small-Cell Neuroendocrine Carcinoma Using a 3D Organoid Model

**DOI:** 10.3390/ijms27052393

**Published:** 2026-03-04

**Authors:** Hasibul Islam Sohel, Umme Farzana Zahan, Masako Ishikawa, Kosuke Kanno, Hitomi Yamashita, Kentaro Nakayama, Satoru Kyo

**Affiliations:** 1 Department of Obstetrics and Gynecology, Faculty of Medicine, Shimane University, Izumo 693-8501, Japan; hasibulsohel1167@gmail.com (H.I.S.); farzanashormi99@gmail.com (U.F.Z.); m-ishi@med.shimane-u.ac.jp (M.I.); kanno39@med.shimane-u.ac.jp (K.K.); meme1103@med.shimane-u.ac.jp (H.Y.); 2 Department of Obstetrics and Gynecology, East Medical Center, Nagoya City University, Nagoya 464-8547, Japan

**Keywords:** cervical cancer, small cell neuroendocrine carcinoma (SCNEC), organoid, spheroid, whole-exome sequencing

## Abstract

Cervical small-cell neuroendocrine carcinoma (SCNEC) is a rare cervical cancer with high metastatic potential and is frequently associated with high-risk human papillomavirus (HPV) infection. Because of its low incidence, SCNEC remains understudied and treatment options are limited, posing major therapeutic challenges. This study aimed to characterize SCNEC at the molecular and functional levels to support more informed therapeutic strategies. Organoids and spheroids were generated from a cervical SCNEC biopsy, and a matched organoid-derived xenograft was established in immunodeficient mice. Model fidelity was evaluated by histopathology and immunohistochemistry. HPV status was assessed by p16 immunostaining and HPV18 PCR, and viral–host integration sites were inferred using whole-exome sequencing (WES) junction reads. WES was also used to compare shared somatic variants and copy-number alterations across the patient tumor, organoid, and xenograft. Drug responses were assessed in organoids and spheroids following exposure to a panel of chemotherapeutic agents and a targeted inhibitor. Organoids exhibited robust growth, morphologic maturation, and efficient recovery after cryopreservation. The organoids and matched xenografts faithfully recapitulated SCNEC, with preserved neuroendocrine differentiation (CD56, synaptophysin, and NSE positivity), a high Ki-67 proliferative index (>80%), and strong p16 expression. HPV18 status was conserved across the primary tumor, organoids, and xenografts, with an integration site at chr8 (8q24.21) associated with increased MYC expression. WES revealed strong cross-model concordance, including 26 shared somatic variants with a canonical *PIK3CA* hotspot mutation (p.E542K) and conserved oncogenic copy-number gains of *PIK3CA*, *TERT*, and *MYC*, as well as copy number loss of *TP53*. Functional assays showed dose-dependent loss of viability following exposure to conventional cytotoxic agents or an mTOR pathway inhibitor. This study presents the first integrated molecular and functional analyses of patient tumors and matched organoid and xenograft models in cervical SCNEC. These models offer robust resources for mechanistic studies and may enable precision therapeutic strategies for this rare malignancy.

## 1. Introduction

Small cell neuroendocrine carcinoma (SCNEC) of the uterine cervix is an exceptionally rare and highly aggressive malignancy. Accounting for only 2–5% of all cervical cancers, yet associated with a markedly poorer prognosis than the two major histological subtypes, squamous cell carcinoma (SCC) and adenocarcinoma (AC) [1,2,3,4,5] for its wide involvement of lymph node (41.6–67%) and metastatic potential at an early stage. As a consequence, the prognosis of SCNEC is far worse than that of the common cervical cancer subtypes, with a 5-year overall survival of nearly 30% compared with more than 65% for SCC and AC [6,7]. According to the 2020 World Health Organization (WHO) classification, cervical neuroendocrine carcinoma (NEC) is divided into two histological variants: small-cell neuroendocrine carcinoma (SCNEC) and large-cell neuroendocrine carcinoma (LCNEC). Between these, SCNEC is more frequently encountered. In Japan, newly diagnosed cervical NEC accounts for approximately 1.6% of all cervical cancers, with SCNEC comprising 1.3% and LCNEC 0.3% of cases [8], consistent with prior epidemiological data [9,10].

Histopathologically, SCNEC is characterized by sheets of small cells with nuclear molding, scant cytoplasm, and extensive necrosis. Immunohistochemical evaluation typically demonstrates expression of classic neuroendocrine markers, including synaptophysin, chromogranin A, and CD56, although expression may be variable [11]. A strong etiological association with high-risk human papillomavirus (HPV) infection has been documented, particularly with HPV18, followed by HPV16, supporting the role of viral oncoproteins in tumorigenesis [11,12,13].

At the molecular level, next-generation sequencing has revealed recurrent genetic alterations in SCNEC. The most frequently reported mutations involve *TP53* (26%), *PIK3CA* (18%), *KRAS* (12%), *RB1* (10%), and *MYC* amplifications (53%), while loss of heterozygosity (LOH) events have been observed in approximately 30% of cases [14,15]. HPV-associated carcinogenesis is a multistep process in which the viral oncoproteins E6 and E7 inactivate p53 and pRb, promoting proliferation and resistance to apoptosis [16,17]. With persistent infection, HPV DNA integration into the host genome can sustain E6/E7 expression and drive additional genomic alterations, including oncogene amplification, chromosomal rearrangements and genomic instability [17]. These molecular observations suggest partial overlapping with small-cell carcinomas of other organs. They also suggest cervix-specific HPV-related mechanisms. However, SCNEC is rare and remains understudied. Molecular profiling linked to functional testing is limited, so the therapeutic vulnerabilities remain unclear.

In this study, a tri-model SCNEC platform was established using patient tumor tissue, 3D organoid culture, and organoid-derived xenografts. This tri-model was used for integrated molecular characterization and functional analysis to better define SCNEC biology and support therapeutic development.

## 2. Results

### 2.1. Development of Organoid Models from the Patient with Cervical SCNEC

**Figure 1:**
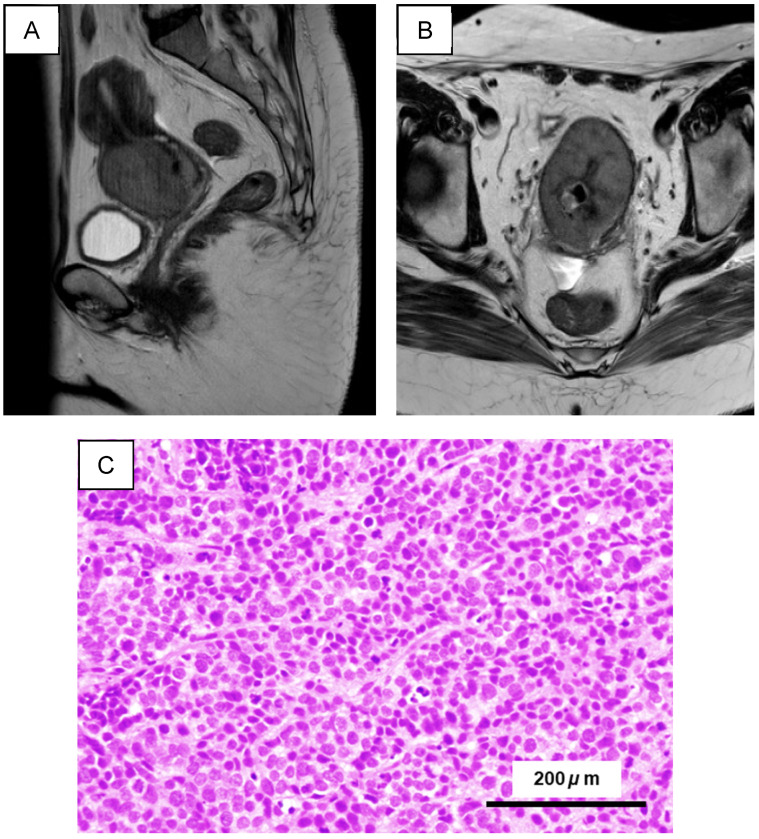
Clinical imaging and histopathology of the cervical SCNEC biopsy. (**A**) Sagittal (side-view) pelvic MRI showing a bulky cervical mass. (**B**) Axial pelvic MRI demonstrating the cervical lesion. (**C**) Representative H&E-stained biopsy section consistent with small-cell neuroendocrine carcinoma. Scale bars, 200 μm.

Biopsy-derived tumor tissue was obtained from a 41-year-old woman diagnosed with a cervical tumor. Pelvic MRI demonstrated a bulky cervical mass consistent with locally advanced disease (Figure 1A,B). Histopathological examination confirmed SCNEC morphology (Figure 1C), characterized by densely packed small cells with scant cytoplasm, nuclear molding, and extensive necrosis. Immunohistochemistry supported the diagnosis, showing CD56 and synaptophysin positivity, chromogranin A negativity, strong and diffuse p16 expression consistent with high-risk HPV-associated disease, and a high proliferative index (Ki-67 ≥ 80%).

**Figure 2:**
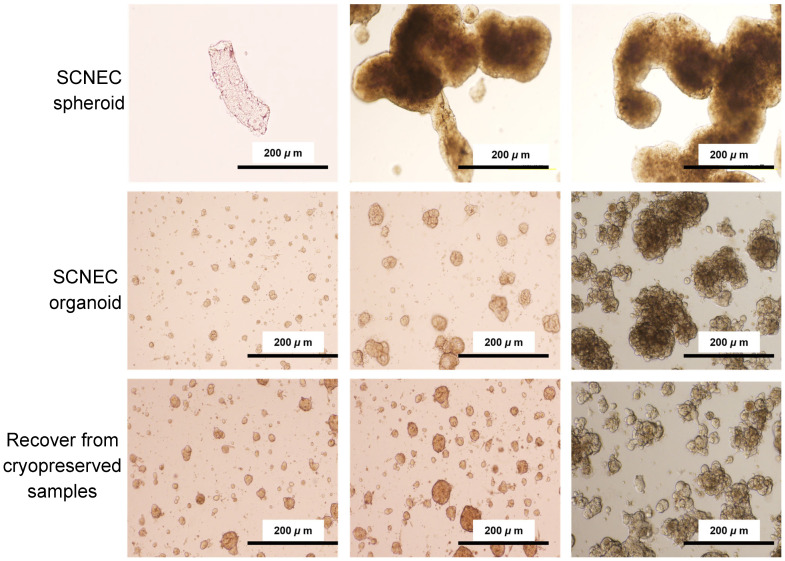
Establishment, expansion, and cryorecovery of cervical SCNEC spheroids and organoids. Representative bright-field images showing biopsy-derived SCNEC spheroids (**top row**), SCNEC organoids during serial expansion in Matrigel (**middle row**), and organoids recovered after cryopreservation and re-culture (**bottom row**). Scale bars, 200 μm.

The patient underwent standard-of-care multimodal treatment, including chemoradiotherapy followed by subsequent systemic therapy during follow-up (Supplementary Table S1). A portion of the specimen was processed for routine diagnostic histopathology, and the remaining tissue was used to establish patient-derived spheroid and organoid cultures as previously described [18,19] in the Materials and Methods. Spheroids were formed as compact multicellular aggregates, while organoids were readily established and expanded in culture (Figure 2). Organoids were also successfully established, demonstrating robust growth with progressive enlargement over time and maintaining stable morphology during serial propagation. Notably, we confirmed that organoids recovered efficiently after cryopreservation and resumed growth promptly upon re-culture (Figure 2), supporting their long-term expandability and suitability for downstream molecular and functional analyses.

### 2.2. Retention of SCNEC-Specific Histological Features in Organoid and Xenograft Models

**Figure 3:**
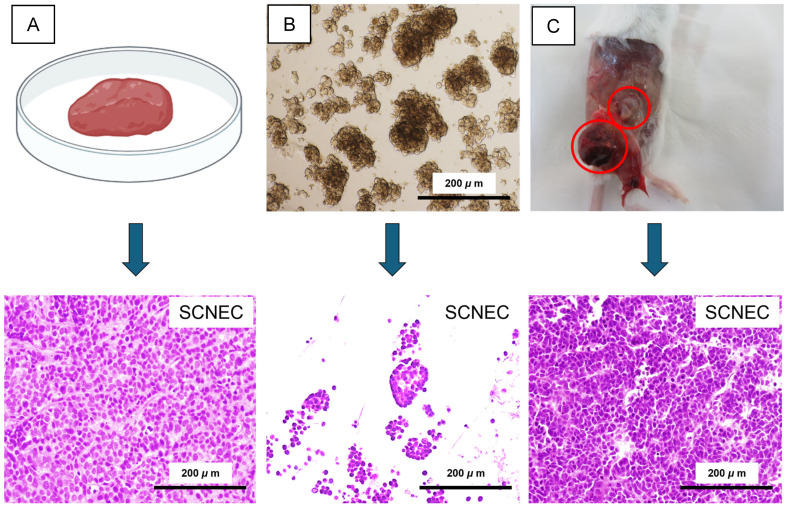
SCNEC histopathology is preserved in patient tumor, organoids, and organoid-derived xenografts. (**A**) Patient biopsy specimen and representative H&E staining showing SCNEC morphology. (**B**) Established SCNEC organoids and corresponding histology demonstrating retention of small-cell features. (**C**) Organoid-derived xenograft tumors (outlined by red circles) in an immunodeficient SCID mouse and matched H&E staining recapitulating SCNEC histology. Scale bars, 200 μm.

Histopathological evaluation of the patient’s cervical biopsy specimen demonstrated features consistent with SCNEC, including nuclear molding, scant cytoplasm, and densely packed tumor cells (Figure 3A). Organoids established from this biopsy retained these morphological characteristics, forming compact structures composed of small, uniform cells with a high nuclear-to-cytoplasmic ratio (Figure 3B). Similarly, organoid-derived xenografts generated in immunodeficient mice reproduced the histological hallmarks of the parental tumor; notably, tumors developed in 4/4 SCID mice, and all retained the defining features of SCNEC (Figure 3C). Collectively, these findings indicate that both organoid and xenograft models recapitulate the histopathological features of SCNEC with high fidelity.

### 2.3. Immunophenotypic Characterization Confirms SCNEC Features in Patient-Derived Models

**Figure 4:**
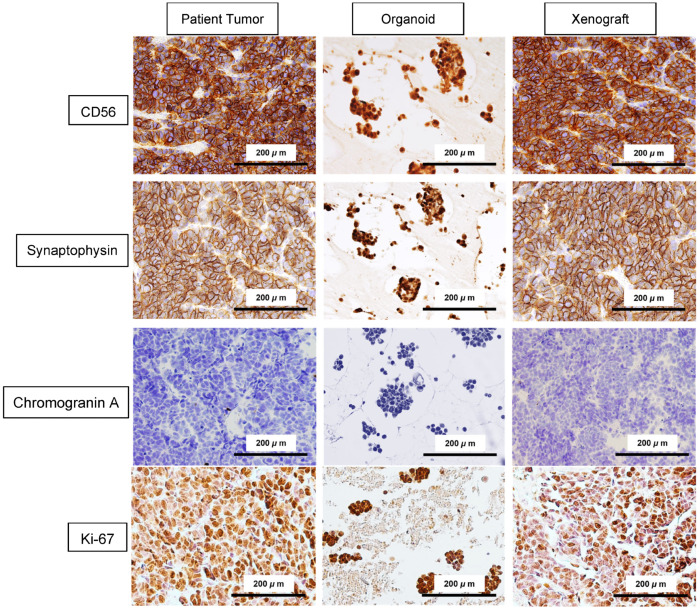

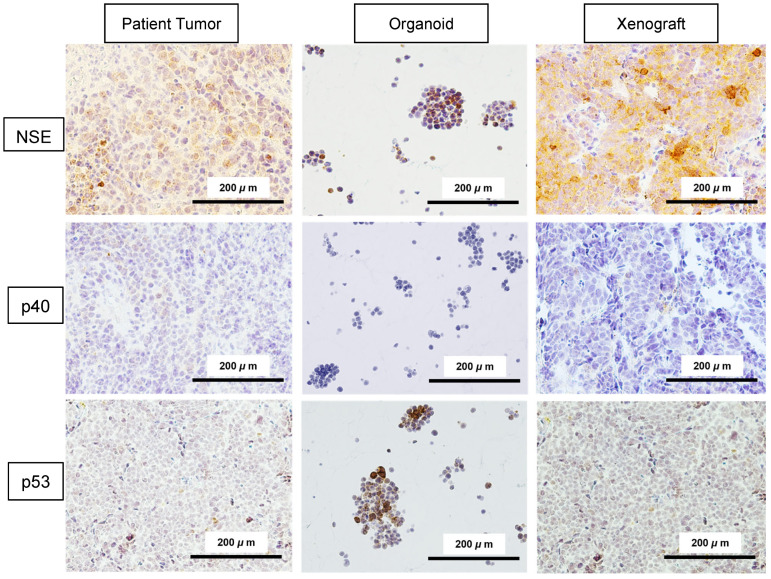
Immunophenotypic validation of SCNEC features in patient tumor, organoids, and organoid-derived xenografts. Representative immunohistochemistry images of neuroendocrine markers (CD56, synaptophysin, and chromogranin A), NSE, proliferation marker Ki-67, squamous marker p40, and p53 in the patient tumor, matched organoids, and xenograft tumors, demonstrating concordant SCNEC immunoprofiles across models. Scale bars, 200 μm.

Immunohistochemistry demonstrated that the patient’s tumor, patient-derived organoids, and organoid-derived xenografts retained a consistent immunophenotype compatible with SCNEC (Figure 4). CD56 showed strong membranous positivity across all three specimens, and synaptophysin exhibited diffuse cytoplasmic staining. The proliferative activity was uniformly high, with a Ki-67 labeling index exceeding 80% and widespread nuclear positivity across the parental tumor and both derived model systems. Neuron-specific enolase (NSE) was moderately positive, whereas chromogranin A was negative throughout. The p40 expression was negative, arguing against squamous differentiation. Notably, p53 protein expression was weak in the patient’s tumor, organoids, and xenografts. Collectively, these findings confirm that the organoid and xenograft models preserve the key features of the parental SCNEC tumor.

### 2.4. Identification of Somatic Mutations and HPV DNAs Across Patient Tumor, Organoid, and Xenograft Models

**Figure 5:**
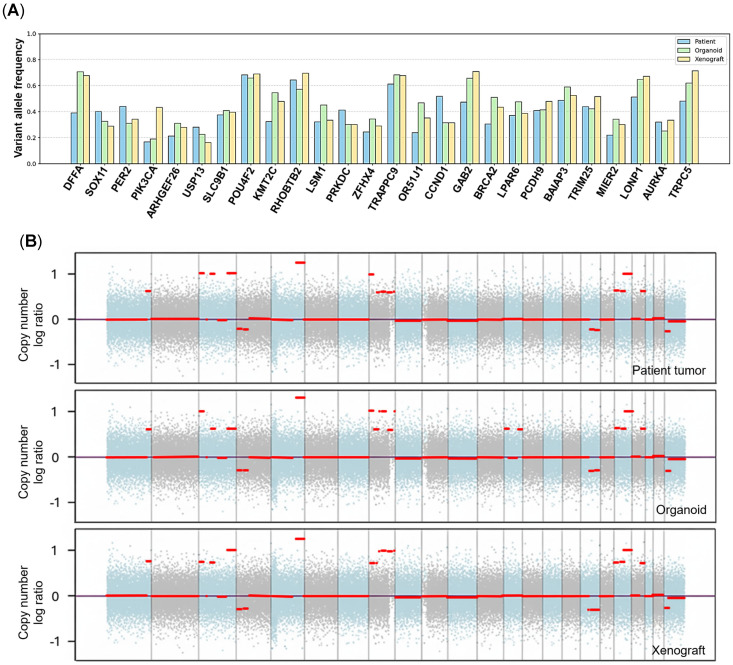
Conserved somatic alterations across the SCNEC tri-model. (**A**) Variant allele frequencies of shared somatic mutations detected by WES in the patient tumor, matched organoids, and organoid-derived xenograft. (**B**) Genome-wide copy-number profiles (log2 ratios) showing largely concordant copy-number alterations across the three models.

To profile mutational status and clonal stability across the SCNEC tri-model, we performed WES of the primary tumor, patient-derived organoid, and matched xenograft, prioritizing protein-altering variants, missense, frameshift insertions/deletions, stop-gain, and splice-site changes (Figure 5A, Table 1, Table 2 and Table 3). The tumor mutation burden was low, consistent with prior observations in neuroendocrine carcinomas [20]. After somatic filtering, 26 somatic variants were shared across all three specimens and showed broadly comparable variant allele frequencies, supporting preservation of the dominant tumor clone during both in vitro culture and in vivo propagation (Figure 5B, Table 1, Table 2 and Table 3). This shared set included the canonical *PIK3CA* p.E542K hotspot alteration, a recurrent driver event in cervical neuroendocrine carcinoma [21].

Among the 26 shared genes, *PIK3CA*, *BRCA2*, *CCND1*, *KMT2C*, *AURKA*, *PRKDC*, and *GAB2* are well-established SCNEC-associated genes catalogued in major resources (OncoKB/COSMIC and TCGA-derived cancer gene curation), whereas many of the additional altered genes are not well characterized in cervical SCNEC and may represent rare or passenger events.

Copy-number analysis identified a broad set of genes with concordant alterations across the patient tumor, derived organoid, and mouse xenograft models. The majority of shared events reflected copy-number gains, with a smaller subset showing copy-number losses, indicating that amplifications are the dominant conserved CNA pattern across the tri-model system. Among the shared alterations, several genes with established relevance to cervical SCNEC and/or neuroendocrine tumor biology were observed, including *MYC*, *TERT*, *TP53*, *AURKA*, *PIK3CA*, *NOTCH3*, and *PRKDC*, consistent with recurrent pathway-level dysregulation in aggressive, high-grade malignancies [20,22]. The remaining shared genes likely include pan-cancer alterations, context-dependent passengers, and some poorly characterized/nonfunctional loci, so they should be interpreted cautiously without expression/functional support (Supplementary Table S2).

**Figure 6:**
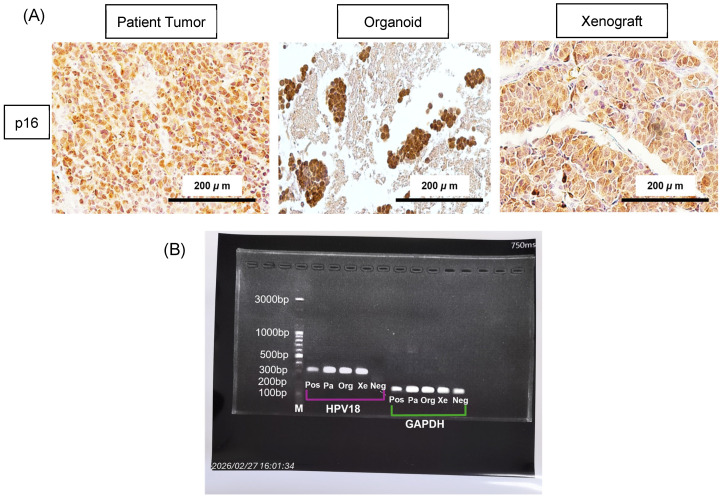

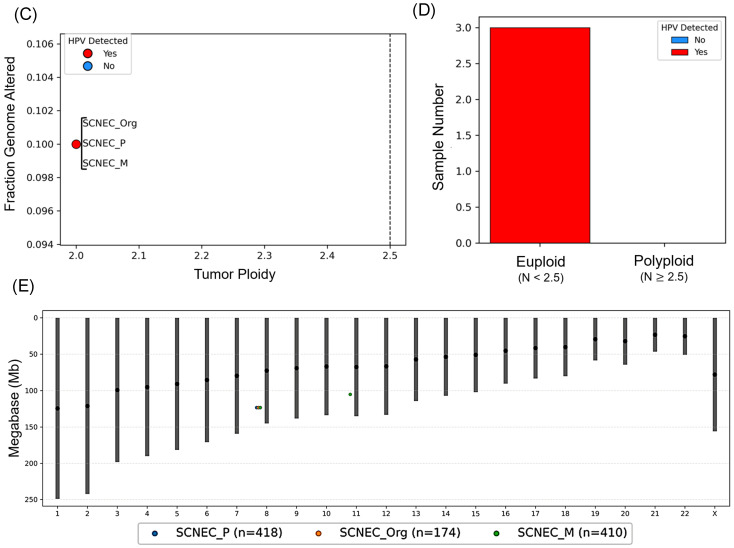
HPV-associated features and HPV18 detection across the SCNEC tri-model. (**A**) p16 immunohistochemistry in the patient tumor, matched organoids, and organoid-derived xenograft showing strong/diffuse staining consistent with HPV-associated disease. (**B**) Type-specific PCR confirming HPV18 DNA in patient tumor (Pa), organoid (Org), and xenograft (Xe); HeLa DNA served as a positive control (Pos), normal cervical tissue as a negative control (Neg), and GAPDH as an input/quality control (M, marker). (**C**) Tumor ploidy versus fraction of genome altered, indicating near-diploid status with comparable genomic alteration across models. (**D**) The presence of HPV is associated with euploid chromosomal content in SCNEC models. (**E**) Genomic positions of HPV18 integration breakpoints inferred from WES viral–host junction reads across the three specimens. SCNEC_P, patient tumor; SCNEC_Org, organoid; SCNEC_M, mouse tumor (Xenograft).

To examine HPV status across the matched SCNEC tri-model (patient tumor, patient-derived organoid, and organoid-derived xenograft), we evaluated p16 immunohistochemistry and HPV18 DNA by type-specific PCR (Figure 6A,B). All three specimens demonstrated strong, diffuse nuclear and cytoplasmic p16 staining, consistent with an HPV-associated phenotype (Figure 6A). Concordantly, HPV18 DNA was amplified from the patient’s tumor and was retained in both the organoid and xenograft using primers targeting the *HPV18 E6/E7* region (Figure 6B). We next leveraged WES to detect viral–host junction reads and map integration events. HPV18 was consistently identified in the patient tumor, matched organoid, and xenograft, with no evidence of multiple HPV subtypes. WES-based copy-number profiling further showed preservation of global genome architecture across the tri-model. Tumor ploidy reflecting the inferred average genome-wide copy-number state remained in the near-diploid/euploid range (ploidy < 2.5, approximating 2N DNA content) in the patient tumor, organoid, and xenograft, with a similar fraction of the genome affected by copy-number alterations (~0.10) (Figure 6C,D). No specimen met criteria for polyploidy (ploidy ≥ 2.5), supporting genomic stability of the culture and engraftment process at the whole-genome level. Integration breakpoints derived from WES junction reads converged on a recurrent hotspot at 8q24.21, clustering near the *MYC* locus across all three specimens, supporting conserved *HPV18* integration targeting of the *MYC* region in this SCNEC tri-model. An additional integration event at 11q14.1 was detected exclusively in the xenograft (Figure 6E).

### 2.5. Drug Response of Patient-Derived SCNEC Organoids and Spheroids to Clinically Relevant Agents

**Figure 7:**
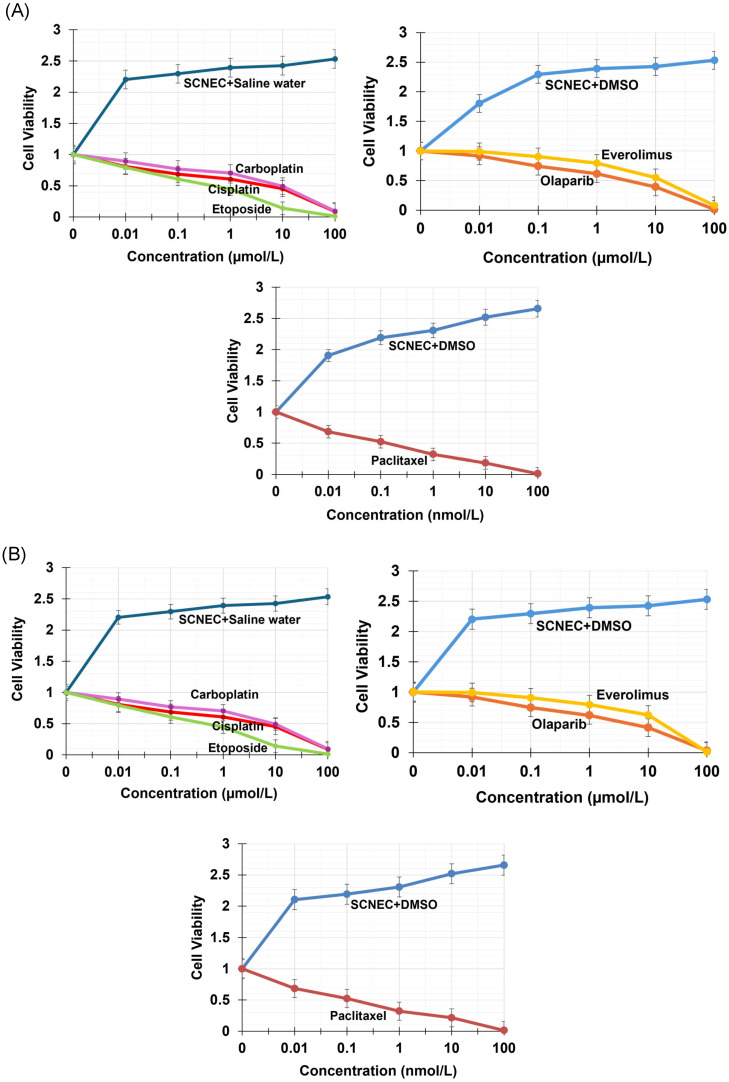
Drug response of patient-derived SCNEC organoids and spheroids. Dose–response curves for clinically relevant agents measured by cell viability assays. (**A**) SCNEC organoids and (**B**) SCNEC spheroids were treated with paclitaxel (SCNEC + DMSO control), cisplatin, carboplatin, etoposide (SCNEC + Saline water control), olaparib, and everolimus (SCNEC + DMSO control) across the indicated concentration ranges, showing dose-dependent growth inhibition after 72 h exposure.

To evaluate the clinical relevance of the patient-derived models, drug sensitivity assays were performed in organoids and spheroids using agents commonly used in cervical cancer and neuroendocrine carcinoma treatment, including a taxane, platinum agents, etoposide, a PARP inhibitor, and an mTOR inhibitor (paclitaxel, cisplatin, carboplatin, etoposide, olaparib, and everolimus). In both models, 24 h exposure produced minimal changes in viability across the tested concentration ranges, whereas 72 h exposure led to clear, dose-dependent reductions in viability in organoids (Figure 7A) and spheroids (Figure 7B). Based on the 72 h dose response curves, the estimated IC_50_ values were approximately 0.34 µM (paclitaxel), 4.36 µM (cisplatin), 4.93 µM (carboplatin), 0.79 µM (etoposide), 1.89 µM (olaparib), and 12.85 µM (everolimus). Overall, both organoids and spheroids showed time-dependent sensitivity to paclitaxel, platinum agents, etoposide, and olaparib, whereas everolimus demonstrated comparatively weaker activity, indicating limited sensitivity under these in vitro conditions.

## 3. Discussion

Cervical neuroendocrine carcinoma is a rare tumor (~1.5% of new cervical cancers) with highly aggressive clinical behavior, with most cases being high-grade small- or large-cell subtypes [14]. Its apparent incidence is rising, yet clinical management remains challenging because prospective trials are scarce and systemic therapy is largely extrapolated from other neuroendocrine malignancies and standard cervical cancer regimens [23]. To address these gaps, we developed a patient-matched tri-model platform including primary tumor, organoid, and organoid-derived xenograft that preserves key diagnostic and molecular hallmarks while enabling functional drug testing. Using WES alongside comparative histopathology, immunoreactivity, and therapeutic evaluation across all three models, this framework provides a tractable way to link HPV-associated oncogenesis and recurrent pathway alterations to treatment response in SCNEC.

We assessed HPV status across all three models because high-risk HPV infection is the main cause of cervical cancer and is frequently reported in cervical neuroendocrine carcinoma [24,25]. Kuji et al. analyzed 37 high-grade cervical neuroendocrine carcinomas (29 small cell and 8 large cell) and found high-risk HPV in 72% of cases, predominantly HPV18 (86%) with fewer HPV16 (14%) [26]. Similarly, Alejo et al. reported HPV positivity in 86% of cervical neuroendocrine tumors, mainly HPV16/18, with other subtypes detected in only 4%, supporting the close association between SCNEC, HPV infection, and p16^INK4a^ overexpression [25]. Consistent with these reports, our patient tumor, organoid, and xenograft showed strong/diffuse p16 expression and were predominantly HPV18-positive, aligning with findings from Xuan Pei et al. and others that HPV18 is particularly related to cervical SCNEC [21,27,28]. Together, these results indicate that our tri-model platform retains HPV-associated biology rather than shifting toward an HPV-independent phenotype during propagation.

Even though SCNEC is as aggressive as small cell carcinoma in other organs (such as small cell lung cancer), its development is distinct because it is linked to human papillomavirus (HPV) infection [29]. HPV-associated cancer follows multistep molecular processes. First, tumorigenesis by high-risk HPVs is driven by their two main viral oncoproteins, E6 and E7, which inactivate p53 and Rb, respectively, leading to cell-cycle deregulation and inhibition of p53-mediated apoptosis [17,30]. In our study, the combination of weak p53 protein expression and *TP53* copy-number loss at 17p supports disruption of the TP53 pathway, consistent with prior reports that, in HPV-driven tumors, p53 levels can be reduced through E6/E6AP-mediated ubiquitin-dependent degradation, and 17p/*TP53* arm loss may further attenuate pathway activity even in the absence of *TP53* mutations [31,32,33,34,35]. Therefore, functional suppression of the p53 axis can occur in HPV-driven SCNEC through viral and genomic mechanisms simultaneously, even when canonical *TP53* point mutations are not detected.

In addition, HPV DNA integration can drive genomic disruption, including oncogene amplification, chromosomal rearrangements, and chromosomal instability [36,37]. MYC overexpression commonly occurs in HPV-associated cervical cancer [38]. In our matched models, the integration mapped to 8q24.21 near MYC, consistent with prior reports identifying this locus as a recurrent hotspot in other cervical cancer subtypes [39]. Notably, HPV18-positive HeLa cells also carry HPV18 integration at 8q24.21, accompanied by increased MYC protein expression [40]. Because *MYC* is key for cell-cycle control, apoptosis, and cellular transformation, HPV integration in this region may disrupt local regulation and give tumor cells a growth advantage, although why this differs by HPV type is still unclear. Prior studies also report that HPV integration often occurs near amplified genomic regions [37,41], so this relationship warrants further investigation.

Finally, the accumulation of DNA alterations in host genes is an important step in the progression from HPV infection to invasive cervical cancer. In genomic analysis of patient tumor, organoid, and xenograft, we observed 26 shared somatic variants, supporting strong genomic fidelity of the matched models. A variety of frequent oncogenic mutations of SCNEC in *PIK3CA*, *BRCA2*, *KMT2C*, *PRKDC*, *AURKA*, *CCND1*, and *SOX11* were detected in all models. The remaining variants (*DFFA*, *ARHGEF26*, *USP13*, *RHOBTB2*, *LSM1*, *ZFHX4*, *TRAPPC9*, *OR51J1*, *GAB2*, *PCDH9*, *MIER2*, *TRPC5*, *SLC9B1*) have reported roles in other cancer-related pathways [20,42,43,44,45,46,47,48,49,50,51,52,53,54,55,56,57,58] but are not widely recognized as core drivers or defining markers of SCNEC, and these variants have not been previously reported in cervical SCNEC.

Among oncogenic mutations detected, *PIK3CA* p.E542K is a canonical hotspot, gain-of-function mutation in the helical domain, and is among the most frequently observed activating *PIK3CA* alterations in human solid tumors; it drives constitutive PI3K signaling with downstream pathway activation [59,60,61,62]. Previous studies have consistently shown a higher prevalence of *PIK3CA* mutations (31.6% in HPV-associated usual-type cervical cancer, 18.18% in HPV-driven SCNEC) than *KRAS* (13.6%) or *TP53* (11.3%) mutations. Prior studies suggest that therapies targeting *PIK3CA* and other altered genes may be promising treatment options for cervical small-cell carcinoma [20,35]. In this setting, everolimus or other inhibitors of the mTOR/PI3K pathway may be considered; however, the value of a *PIK3CA* mutation alone for predicting response to single-agent everolimus is uncertain, especially when multiple genetic alterations are present [63,64], underscoring why patient-matched functional testing is valuable.

Copy-number analysis showed many genes with matching alterations in our study across the patient tumor, derived organoid, and mouse xenograft models. High levels of CNAs likely cooperate with HPV integration-associated genomic instability to accelerate clonal evolution and progression in HPV-driven cancers [41,65,66,67]. We identified multiple oncogenic CNA events affecting key cancer pathways, including *TERT*, *PIK3CA*, *MYC,* and *NOTCH3* amplifications, as well as *TP53* copy-number loss, supporting potential activation of telomerase, PI3K/AKT signaling, MYC or Notch programs, and disruption of the p53 pathway. These pathways are recurrently implicated in high-grade neuroendocrine carcinoma of the cervix [20,22,68,69]. *TERT* amplification is a recognized route to telomerase reactivation, supporting telomere maintenance and replicative immortality during tumor evolution. In neuroendocrine malignancies, increased TERT gene dosage correlates with higher TERT expression and has been linked to adverse outcomes, suggesting that TERT activation may contribute to aggressive clinical behavior. Accordingly, the *TERT* amplification observed in our HPV-associated SCNEC models represents a biologically plausible cooperating event that may facilitate progression alongside HPV-driven oncogenesis and widespread copy-number changes.

At the phenotypic level, our immunohistochemistry demonstrated robust phenotypic fidelity across the patient tumor, organoid, and xenograft. The neuroendocrine immunophenotype was conserved, with CD56 and synaptophysin positivity and chromogranin A negativity, a pattern frequently described in cervical SCNEC, where chromogranin is specific but variably expressed. NSE positivity and p40 negativity were similarly preserved, reinforcing that the matched models maintain the defining diagnostic features of SCNEC [14,25,70,71]. This concordance is important because it confirms that the tri-model platform preserves both molecular events and the diagnostic phenotype that defines SCNEC clinically.

Importantly, the organoid- and spheroid-based platforms also enable functional therapeutic testing in a patient- and SCNEC-specific manner, linking drug response to the underlying molecular profile. By integrating HPV status, viral-host genomic features, recurrent pathway alterations, and matched drug testing, this tri-model approach provides a practical framework for studying treatment vulnerabilities in rare diseases where evidence is limited and standard regimens are therefore extrapolated. However, this tri-model platform represents one patient’s tumor biology; therefore, the frequency of the observed variants/CNAs and the generalizability of drug-response patterns to broader SCNEC populations should be further investigated with additional SCNEC patients and tri-models.

## 4. Materials and Methods

### 4.1. Clinical Sample and Patient Information

Biopsy specimens were obtained from a 41-year-old female patient diagnosed with small cell neuroendocrine carcinoma (SCNEC) of the uterine cervix. The diagnosis was confirmed by experienced pathologists through histopathological evaluation at Shimane University Hospital. All experimental procedures were approved by the Ethics Committee of Shimane University Faculty of Medicine (IRB No. 20070305-1 and 20070305-2). Written informed consent was obtained from the patient for the use of her clinical and pathological specimens in this study. All methods were carried out in accordance with the ethical principles of the Declaration of Helsinki.

### 4.2. Generation and Maintenance of Primary Tumor Organoids

Fresh surgical biopsy tissue from a patient with small cell neuroendocrine carcinoma of the cervix (SCNEC) was processed for organoid culture. The specimen was cut into 2–3 mm fragments, washed several times with ice-cold phosphate-buffered saline (PBS), and enzymatically dissociated into small clusters or single cells using dispase II (2 U/mL; Wako, Osaka, Japan), collagenase P (1 mg/mL; Roche Diagnostics GmbH, Mannheim, Germany), and DNase I (10 U; Sigma-Aldrich, St. Louis, MO, USA) for 45 min at 37 °C. The partially digested tissue was further treated with Accumax (Life Technologies Corporation, Grand Island, NY, USA) for 5 min at 37 °C to achieve a single-cell suspension. When residual red blood cells were present, ACK (Life Technologies Corporation, Grand Island, NY, USA) lysing buffer was applied for 5 min at 37 °C, followed by washing with ice-cold PBS. A total of 3 × 10^6^ viable cells were resuspended in organoid culture medium (Supplementary Table S3) (800 μL/well) and seeded onto a solidified layer of Matrigel (65 μL/well; Discovery Labware, Inc., Bedford, MA, USA) in 12-well plates. After overnight incubation at 37 °C, attached cells were overlaid with an additional 70 μL of Matrigel and supplemented with 800 μL/well of fresh medium. Cultures were maintained at 37 °C, and the medium was replaced every 2–3 days.

Organoids were passaged upon reaching 70–80% confluency, typically at a 1:4 to 1:6 dilution. For passaging, organoids embedded in Matrigel and culture medium were collected using a cell scraper, washed with PBS, and dissociated into single cells by Accumax treatment (5 min, 37 °C) with gentle pipetting before reseeding.

### 4.3. Spheroid Culture

Spheroids were established from SCNEC patient tumor tissue following our recently published study [18]. Briefly, well-preserved single glands were isolated under a stereomicroscope and cultured together in a 6-well non-adherent dish in a humidified incubator (37 °C, 5% CO_2_). Cultures were maintained for up to 3 months with medium changes every 3–4 days.

### 4.4. Assay for Tumorigenicity

Immunocompromised female C.B-17/Icr-scid/scidJc1 (CLEA Japan, Inc., Shizuoka, Japan) SCID mice aged 5–7 weeks were used for this study. Mice were acclimatized for 4 weeks before experiments. All animal procedures were approved by the Ethics Committee for Animal Experimentation of Chiba Cancer Center and the Animal Care and Use Committee of Shimane University and conducted in accordance with institutional and national guidelines. Tumor-derived organoids (5 × 10^5^ cells) were resuspended in 100 μL advanced DMEM/F12 and mixed with 100 μL Matrigel (1:1 ratio) before subcutaneous injection into the right dorsum of three SCID mice. Tumor growth was monitored twice weekly by inspection and palpation. After 3–4 months, tumors reached a sufficient size for further experiments. Tumors were fixed in 4% buffered formaldehyde, and sections were stained with hematoxylin and eosin (H&E) for histological analysis.

### 4.5. Pathological Evaluation

The original tumor, organoids, and organoid-derived xenografts were fixed with 4% neutral buffered formalin. They were then dehydrated, embedded in paraffin, and sectioned at 5 μm [72,73] for the organoid and organoid-derived xenografts (Supplementary Methods S1 and S2). The sections were deparaffinized and stained with hematoxylin and eosin. Primary antibodies against the following proteins were used for immunohistochemical studies of the original tumor, organoids, and organoid-derived xenografts: CD56, synaptophysin, chromogranin A, NSE, p40, p16, Ki-67, and p53 (Supplementary Table S4).

### 4.6. Analysis of Genetic Aberration

Genomic DNA was extracted from formalin-fixed paraffin-embedded (FFPE) tumor tissue, SCNEC organoid, and organoid-derived xenograft using the QIAamp DNA FFPE Tissue Kit (Qiagen, Hilden, Germany) according to the manufacturer’s protocol and previous studies [74]. The Agilent 2000 Tape Station (Agilent Technologies, Santa Clara, CA, USA) was first used to assess DNA integrity. Subsequently, WES using enriched amplicons was performed on the Illumina MiSeq (San Diego, CA, USA). The sequencing data were analyzed using the Human WES Standard Analysis bioinformatics pipeline (Filgen Inc., Nagoya, Japan). High analytical sensitivity and specificity were ensured throughout the investigation through the use of sequence alignment, variant calling, variant filtering, variant annotation, and variant prioritization. Tumor purity and ploidy estimates were derived with Sequenza (v3.0.0) from B-allele frequency (BAF) distributions and copy-number segmentation profiles [75].

### 4.7. Somatic Variant Analysis

Sequence reads were aligned to the hg38 reference and quality-filtered and trimmed using fastp (v0.23.1) and aligned to the hg38 reference genome with BWA (v0.7.17). After sorting and duplicate marking (sambamba v1.0.0), germline SNPs/Indels were called using GATK (v4.3.0). Somatic variants were identified from tumor–normal paired analysis using MuTect (v2.2-25) and Strelka (v2.9.10), defining somatic mutations as variants present in tumor DNA but absent in matched normal DNA. Variants were annotated using ANNOVAR (v2019Oct24), and copy number variations were inferred using Control-FREEC (v11.4) based on read-depth/log2 ratio profiles: >0.3 for amplifications, <−0.3 for deletions.

### 4.8. HPV DNA Detection

HPV18 DNA was detected by nested, type-specific conventional PCR targeting the E6/E7 region, which is commonly retained in HPV-associated cancers and is suitable for FFPE-derived DNA. Genomic DNA was extracted from representative FFPE tumor sections using standard procedures. In the first round, broad HPV amplification was performed using GP-E6/E7 consensus primers as described previously [76]. The second (nested) round was performed using HPV18 type-specific E6/E7 primers: HPV18-F (5′-CACTTCACTGCAAGACATAGA-3′) and HPV18-R (5′-GTTGTGAAATCGTCGTTTTTC A-3′), yielding an expected amplicon of around 322 bp [77]. DNA integrity and PCR amplifiability were verified by parallel amplification of GAPDH (Forward: 5′-TGTTGCCATCAATGACCCCTT-3′; Reverse: 5′-CTCCACGACGTACTCAGCG-3′) as an internal control. PCR reactions were performed in a 20 µL volume containing ~30 ng template DNA (first round) or 1 µL of diluted first-round product (e.g., 1:10–1:50 dilution) for the nested round, along with standard buffer, MgCl_2_, dNTPs, primers, and DNA polymerase. Cycling conditions were: 94 °C for 2 min, 42 cycles of 94 °C for 30 s, 56 °C for 30 s, extension at 70 °C for 30 s, and 72 °C for 1 min, with a final extension at 70 °C for 5 min. Amplicons were resolved on a 2% agarose gel and visualized under UV illumination. Samples were considered HPV18-positive when the expected HPV18 E6/E7 band was detected together with a valid GAPDH band. An HPV18-positive control and a no-template control were included in each run.

### 4.9. HPV Integration Site Detection from WES

To detect HPV-derived sequences in WES data, we constructed a custom reference genome by concatenating the human reference genome (hg38) with NCBI reference genomes for HPV16, HPV18, HPV31, HPV33, HPV45, HPV51, and HPV52. After adapter/quality preprocessing, paired-end reads were aligned to the combined hg38/HPV reference using the same alignment pipeline described above, and BAM files were generated following standard post-alignment processing. Reads aligning to HPV reference contigs were summarized to assess HPV presence and coverage, and regions of enriched HPV-aligned read depth were optionally identified using MACS2 [78]. To infer HPV integration breakpoints, structural variant calling was performed on the hg38/HPV-aligned BAMs using three independent tools: DELLY, LUMPY, and Break Dancer [79,80,81], which leverage discordant read pairs and split-read evidence. A candidate HPV integration event was considered high confidence when at least two of the three callers reported a concordant human HPV fusion breakpoint mapping to the same chromosomal locus.

### 4.10. Assays for Drug Sensitivity

Organoids were collected and dissociated into single cells for the in vitro experiment by digesting with Accumax. A TC20 Automated Cell Counter (BioRad, Hercules, CA, USA) was used to count the dissociated cells. For drug sensitivity testing, 4 × 10^3^ single cells/well were plated into PrimeSurface 96U (Sumitomo Bakelite, Tokyo, Japan) in triplicate. At 48 h after plating, paclitaxel (Toronto Research Chemicals Inc., Toronto, ON, Canada), cisplatin (Nichi-Iko Pharmaceutical Co., Ltd., Toyama, Japan), carboplatin (Fujifilm Wako Pure Chemical Corporation, Osaka, Japan) etoposide (E1383-25MG, Sigma-Aldrich, St. Louis, MO, USA), and everolimus (LC Laboratories, Woburn, MA, USA) were added in five serially diluted doses from 0.1 to 100 nM, from 0.1 to 100 μM, from 0.1 to 100 μM, from 0.1 to 100 μM, and from 0.1 to 100 μM, respectively. Cell viability was analyzed using Cell Titer-Glo 3D (Promega, Fitchburg, WI, USA) in triplicate after 72 h of drug incubation. The half-maximal inhibitory concentration (IC50) was calculated by GraphPad Prism software.

### 4.11. Statistical Analysis

The statistical analyses were performed using GraphPad Prism 6 (GraphPad Software, San Diego, CA, USA). The statistical significance was assessed using one-way ANOVA followed by Tukey’s test for multiple comparisons.

## 5. Conclusions

In summary, we established a patient-matched tri-model system that preserves defining diagnostic and molecular hallmarks of cervical SCNEC, enabling functional drug testing. To our knowledge, comprehensive integration of these three patient-matched platforms has been rarely reported in this tumor. The models retained HPV-associated biology with strong/diffuse p16 expression and predominant HPV18, demonstrating concordant alterations spanning viral-host features, shared somatic variants, and recurrent copy-number changes affecting key oncogenic pathways. These findings support the utility of this platform as a tractable experimental framework to connect HPV-driven oncogenesis and pathway alterations with therapeutic vulnerability. More broadly, this approach offers a practical strategy to study rare aggressive malignancies.

## Figures and Tables

**Table 1:** WES summary of somatic mutations in the patient’s tumor.

Gene	ExonicFunc	Protein	VAF	CNA	CNV Type	COSMIC ID	ClinVar
*DFFA*	missense SNV	p.E143Q	0.388888889	3	gain	527327	
*SOX11*	missense SNV	p.D436N	0.4	2	neutral		
*PER2*	missense SNV	p.R952W	0.44	2	neutral	7618085	
*PIK3CA*	missense SNV	p.E542K	0.166666667	4	Amp	5269	P
*ARHGEF26*	missense SNV	p.D328H	0.212121212	4	Amp		
*USP13*	missense SNV	p.E98G	0.28	4	Amp		
*SLC9B1*	frameshift deletion	p.L447Rfs*47	0.375	1	Loss		P
*POU4F2*	non-frameshift insertion	p.G68_R69insG	0.682758621	1	Loss		
*KMT2C*	stop gain	p.Y816*	0.322580645	2	neutral		B
*RHOBTB2*	missense SNV	p.T55M	0.643312102	3	gain	9560360	LB
*LSM1*	missense SNV	p.E110Q	0.321428571	3	gain		
*PRKDC*	splicing		0.411764706	3	gain		B
*ZFHX4*	missense SNV	p.E2718K	0.242718447	3	gain	3716042	
*TRAPPC9*	missense SNV	p.E499Q	0.611764706	3	gain	6563356	
*OR51J1*	missense SNV	p.E94K	0.237288136	2	neutral	4193759	
*CCND1*	UTR3	c.*2137_*2138insT	0.517241379	2	neutral		
*GAB2*	missense SNV	p.Q194H	0.472972973	2	neutral		
*BRCA2*	missense SNV	p.E2175K	0.304347826	2	neutral		VUS
*LPAR6*	missense SNV	p.K125N	0.370967742	2	neutral	8248159	
*PCDH9*	missense SNV	p.H1020Y	0.408450704	2	neutral		
*BAIAP3*	missense SNV	p.G208S	0.486111111	2	neutral		
*TRIM25*	missense SNV	p.E474K	0.4375	2	neutral		
*MIER2*	missense SNV	p.I288M	0.219653179	4	Amp	7119042	
*LONP1*	missense SNV	p.E535K	0.512437811	3	gain	4855663	VUS
*AURKA*	missense SNV	p.E260Q	0.32	3	gain		
*TRPC5*	stop gain	p.E967X	0.480769231	1	Loss		

ExonicFunc, exonic function; VAF, variant allele frequency; CNA, copy number alteration; CNV, copy number variation; Amp, amplification; P, pathogenic; B, benign; LB, likely benign; VUS, variant of uncertain significance; *, indicating stop codon number.

**Table 2:** WES summary of somatic mutations in the organoid.

Gene	ExonicFunc	Protein	VAF	CNA	CNV Type	COSMIC ID	ClinVar
*DFFA*	missense SNV	p.E143Q	0.705882353	3	gain	527327	
*SOX11*	missense SNV	p.D436N	0.325581395	2	neutral		
*PER2*	missense SNV	p.R952W	0.30952381	2	neutral	7618085	
*PIK3CA*	missense SNV	p.E542K	0.18974359	4	Amp	5269	P
*ARHGEF26*	missense SNV	p.D328H	0.30952381	3	gain		
*USP13*	missense SNV	p.E98G	0.225806452	4	Amp		
*SLC9B1*	frameshift deletion	p.L447Rfs*47	0.409090909	1	Loss		P
*POU4F2*	non-frameshift insertion	p.G68_R69insG	0.657446809	1	Loss		
*KMT2C*	stop gain	p.Y816*	0.545454545	2	neutral		B
*RHOBTB2*	missense SNV	p.T55M	0.571428571	3	gain	9560360	LB
*LSM1*	missense SNV	p.E110Q	0.450980392	4	Amp		
*PRKDC*	splicing		0.3	4	Amp		B
*ZFHX4*	missense SNV	p.E2718K	0.34375	4	Amp	3716042	
*TRAPPC9*	missense SNV	p.E499Q	0.681818182	3	gain	6563356	
*OR51J1*	missense SNV	p.E94K	0.466666667	2	neutral	4193759	
*CCND1*	UTR3	c.*2137_*2138insT	0.31372549	2	neutral		
*GAB2*	missense SNV	p.Q194H	0.657142857	2	neutral		
*BRCA2*	missense SNV	p.E2175K	0.510204082	3	gain		VUS
*LPAR6*	missense SNV	p.K125N	0.474576271	3	gain	8248159	
*PCDH9*	missense SNV	p.H1020Y	0.414634146	2	neutral		
*BAIAP3*	missense SNV	p.G208S	0.589473684	2	neutral		
*TRIM25*	missense SNV	p.E474K	0.421568627	2	neutral		
*MIER2*	missense SNV	p.I288M	0.341176471	3	gain	7119042	
*LONP1*	missense SNV	p.E535K	0.64806867	3	gain	4855663	VUS
*AURKA*	missense SNV	p.E260Q	0.25	3	gain		
*TRPC5*	stop gain	p.E967X	0.619047619	1	Loss		

ExonicFunc, exonic function; VAF, variant allele frequency; CNA, copy number alteration; CNV, copy number variation; Amp, amplification; P, pathogenic; B, benign; LB, likely benign; VUS, variant of uncertain significance; *, indicating stop codon number.

**Table 3:** WES summary of somatic mutations in the xenograft.

Gene	ExonicFunc	Protein	VAF	CNA	CNV Type	COSMIC ID	ClinVar
*DFFA*	missense SNV	p.E143Q	0.676470588	3	gain	527327	
*SOX11*	missense SNV	p.D436N	0.287313433	2	neutral		
*PER2*	missense SNV	p.R952W	0.342519685	2	neutral	7618085	
*PIK3CA*	missense SNV	p.E542K	0.432653061	4	Amp	5269	P
*ARHGEF26*	missense SNV	p.D328H	0.278688525	3	gain		
*USP13*	missense SNV	p.E98G	0.161290323	3	gain		
*SLC9B1*	frameshift deletion	p.L447Rfs*47	0.394736842	1	Loss		P
*POU4F2*	non-frameshift insertion	p.G68_R69insG	0.69028777	1	Loss		
*KMT2C*	stop gain	p.Y816*	0.47826087	2	neutral		B
*RHOBTB2*	missense SNV	p.T55M	0.695883134	4	Amp	9560360	LB
*LSM1*	missense SNV	p.E110Q	0.333333333	4	Amp		
*PRKDC*	splicing		0.3	3	gain		B
*ZFHX4*	missense SNV	p.E2718K	0.288951841	4	Amp	3716042	
*TRAPPC9*	missense SNV	p.E499Q	0.676923077	4	Amp	6563356	
*OR51J1*	missense SNV	p.E94K	0.351648352	2	neutral	4193759	
*CCND1*	UTR3	c.*2137_*2138insT	0.31372549	2	neutral		
*GAB2*	missense SNV	p.Q194H	0.707792208	2	neutral		
*BRCA2*	missense SNV	p.E2175K	0.434782609	2	neutral		VUS
*LPAR6*	missense SNV	p.K125N	0.386666667	2	neutral	8248159	
*PCDH9*	missense SNV	p.H1020Y	0.479166667	2	neutral		
*BAIAP3*	missense SNV	p.G208S	0.524663677	2	neutral		
*TRIM25*	missense SNV	p.E474K	0.514905149	2	neutral		
*MIER2*	missense SNV	p.I288M	0.30095037	4	Amp	7119042	
*LONP1*	missense SNV	p.E535K	0.670909091	3	gain	4855663	VUS
*AURKA*	missense SNV	p.E260Q	0.333333333	3	gain		
*TRPC5*	stop gain	p.E967X	0.714285714	1	Loss		

ExonicFunc, exonic function; VAF, variant allele frequency; CNA, copy number alteration; CNV, copy number variation; Amp, amplification; P, pathogenic; B, benign; LB, likely benign; VUS, variant of uncertain significance; *, indicating stop codon number.

## Data Availability

The original contributions presented in this study are included in the article/Supplementary Materials. Further inquiries can be directed to the corresponding author.

## Supplementary Materials

The following supporting information can be downloaded at: https://www.mdpi.com/article/10.3390/ijms27052393/s1.

## Abbreviations

The following abbreviations are used in this manuscript:

SCNEC	Small-cell neuroendocrine carcinoma
LCNEC	Large-cell neuroendocrine carcinoma
SCC	Squamous cell carcinoma
AC	Adenocarcinoma
HPV	Human papillomavirus
WES	Whole-exome sequencing
CNA	Copy number alteration
UTR3	3′ untranslated region
