# Supplementary material for: Integrated Molecular and Functional Characterization of Cervical Small-Cell Neuroendocrine Carcinoma Using a 3D Organoid Model
Source: Int J Mol Sci. 2026 Mar 4;27(5):2393. doi: 10.3390/ijms27052393 (PMC12986295; doi:10.3390/ijms27052393)
Supplement: Supplementary file 1 [file ijms-27-02393-s001.zip › Supplementary method.pdf]

*Supplementary Materials*

# Integrated Molecular and Functional Characterization of Cervical Small-Cell Neuroendocrine Carcinoma Using a 3D Organoid Model

**Hasibul Islam Sohel [1], Umme Farzana Zahan [1], Masako Ishikawa [1], Kosuke Kanno [1], Hitomi Yamashita [1], Kentaro Nakayama [2,*] and Satoru Kyo [1,*]**

**Supplementary method S1:** SCNEC organoid fixation and FFPE block preparation

Organoid culture medium was removed and replaced with advanced DMEM. Plates were placed on ice to liquefy the Matrigel, and organoids were detached using Accumax (5 min, 37 °C), followed by washing with ice-cold PBS. Organoids were then transferred to 5 mL screw-cap cryotubes (Sarstedt, Ref. #60.9921.524) with 4% PFA and kept at room temperature (RT) for 20 min. Samples were centrifuged at 500 g for 5 min at 4 °C, and pellets were washed twice with PBS. Organoids were fixed with 4% paraformaldehyde (PFA) in PBS for 2 h at RT, followed by centrifugation (500 g, 5 min, RT) and removal of fixative.

A 2% agarose gel (Thermo Scientific, Cat. #HG-4000-012) in PBS was prepared, melted, and cooled to 60 °C before being added (200 μL) to the organoid pellets. The mixture was allowed to solidify at RT for 30 min or at −20 °C for 10 min, then transferred to tissue cassettes with a sponge to retain small organoids. Samples were processed through a graded ethanol series (70%, 80%, 90%, 100% I & II), xylene (I & II), and paraffin (I & II), each for 30 min, and finally embedded in paraffin to generate FFPE blocks.

**Supplementary method S2**: SCNEC organoid-derived mouse tumor fixation and FFPE block preparation

The first tumor was isolated from mice. Then, cut 1/3 of the tumor for FFPE block making. The tumor sample was transferred into a screw-cap glass bottle with PFA and kept at room temperature (RT) for 24 hours. Samples were processed through a graded ethanol series (70%, 80%, 90%, 100% I & II), xylene (I & II), and paraffin (I & II), each for 2-3 hours, and finally embedded in paraffin to generate FFPE blocks.
